# Infection by Diverse HIV-1 Subtypes Leads to Different Elevations in HERV-K Transcriptional Levels in Human T Cell Lines

**DOI:** 10.3389/fmicb.2021.662573

**Published:** 2021-05-17

**Authors:** Xi Li, Yaolin Guo, Hanping Li, Xiaofeng Huang, Zhichao Pei, Xiaolin Wang, Yongjian Liu, Lei Jia, Tianyi Li, Zuoyi Bao, Xiaorui Wang, Leilei Han, Jingwan Han, Jingyun Li, Lin Li

**Affiliations:** ^1^State Key Laboratory of Pathogen and Biosecurity, Department of AIDS Research, Beijing Institute of Microbiology and Epidemiology, Beijing, China; ^2^The Second Medical Center, National Clinical Research Center for Geriatric Diseases, Chinese PLA General Hospital, Beijing, China; ^3^Department of Microbiological Laboratory Technology, School of Public Health, Cheeloo College of Medicine, Shandong University, Key Laboratory of Infectious Disease Control and Prevention in Universities of Shandong, Jinan, China; ^4^School of Public Health and Affiliated Shijiazhuang Fifth Hospital, North China University of Science and Technology, Tangshan, China

**Keywords:** HERV-K (HML-2), HIV-1, RNAscope, Tat, CRF01_AE

## Abstract

Human endogenous retroviruses (HERVs) make up ~8% of the human genome, and for millions of years, they have been subject to strict biological regulation. Many HERVs do not participate in normal physiological activities in the body. However, in some pathological conditions, they can be abnormally activated. For example, HIV infection can cause abnormal activation of HERVs, and under different infection conditions, HERV expression may be different. We observed significant differences in HERV-K transcription levels among HIV-1 subtype-infected individuals. The transcriptional levels in the HERV-K *gag* region were significantly increased in HIV-1 B subtype-infected patients, while the transcriptional levels in the HERV-K *pol* region were significantly increased in CRF01_AE and CRF07_BC subtype-infected patients. *In vitro*, the transcriptional levels of HEVR-K were increased 5-fold and 15-fold in MT2 cells transfected with two different HIV-1 strains (B and CRF01_AE, respectively). However, there was no significant difference in transcriptional levels among regions of HERV-K. When MT2 cells were infected with different subtypes of HIV-1 Tat proteins (B, CRF01_AE), which is constructed by lentiviruses, and the transcription levels of HERV-K were increased 4-fold and 2-fold, respectively. Thus, different subtypes of HIV-1 have different effects on HERV-K transcription levels, which may be caused by many factors, not only Tat protein.

## Introduction

Human endogenous retroviruses (HERVs), which infected and integrated into the human genome tens of millions of years ago, account for 8% of the total human genome and can be inherited by future generations (Hughes and Coffin, 2001; Lander et al., 2001). The human genome project revealed that HERV-K is the most biologically specific form of an endogenous retrovirus, but most of the genes of HERV-K members are defective due to mutations that impair their ability to form infectious virus particles (Hughes and Coffin, 2001; Lander et al., 2001; Grandi et al., 2017; Garcia-Montojo et al., 2018; White et al., 2018; Pisano et al., 2019). Members of HERV-K that can replicate may cause disease if activated (Garcia-Montojo et al., 2018). In recent years, it was found that the abnormal expression of HERV-K protein and reverse transcriptional elements may lead to the development of pathological conditions (Qadir et al., 2017; Grandi and Tramontano, 2018; Küry et al., 2018; Matteucci et al., 2018; Kaplan et al., 2019; Alcazer et al., 2020; Curty et al., 2020; Salavatiha et al., 2020). HML-2 is the most active member of the HERV-K family and has the shortest time to integrate into the human genome. RNA expression and protein products of HERV-K (HML-2) have been found in a variety of autoimmune diseases and malignancies (Evans et al., 2009; Serafino et al., 2009; Tai et al., 2009; Reiche et al., 2010; Downey et al., 2015; Küry et al., 2018; Matteucci et al., 2018; White et al., 2018; Levet et al., 2019; Alcazer et al., 2020; Curty et al., 2020; Salavatiha et al., 2020). Despite an increasing number of these reports, no causal relationship has been identified (Hurst et al., 2016; Qadir et al., 2017; Bannert et al., 2018; Matteucci et al., 2018; White et al., 2018; Kaplan et al., 2019; Levet et al., 2019; Curty et al., 2020; Salavatiha et al., 2020). Some pathological conditions may be related to the upregulation of RNA transcription of HERV-K (HML-2), the irregular expression of viral proteins and viral particles, or the production of autoantibodies against HERV-K (Jones et al., 2012; Hurst et al., 2016; White et al., 2018). The HERV-K (HML-2) proteins Rec and Np9 provide a potential link between HERV-K (HML-2) and oncogenesis (Demarchi et al., 1996; Armbruester et al., 2002, 2004; Büscher et al., 2006; Denne et al., 2007; Kaufmann et al., 2010; Gonzalez-Hernandez et al., 2012; Bhardwaj et al., 2014). Both proteins have been shown to stimulate c-Myc expression by binding and inhibiting the c-myc gene repressor promyelocytic leukemia zinc-finger protein, and Rec has also recently been shown to interact with testicular zinc-finger protein, another transcriptional repressor (Denne et al., 2007; Kaufmann et al., 2010).

Some studies have shown that HERV-K (HML-2) can increase its copy number in cells through other virus infections, which also proves the existence of some replication-competent HERV-K proviruses in the human genome (Bhardwaj et al., 2014, 2015). HIV-1 is a human exogenous retrovirus, and many works in the literature have reported that the reverse transcriptional elements of HIV-1 may be universal with HERV-K (Brinzevich et al., 2014; Contreras-Galindo et al., 2017; Young et al., 2018; Gray et al., 2019; Srinivasachar Badarinarayan et al., 2020). Anti-HERV-K antibodies can be detected in 70% of HIV-1-infected patients (Löwer et al., 1996; Büscher et al., 2006; Garson et al., 2019). HERV-K transcription levels are increased in patients diagnosed with HIV-1 encephalopathy (Johnston et al., 2001; Garson et al., 2019). All of these reports indicate that there are some mechanisms shared between HIV-1 and HERV-K in HIV-infected people that affect the transcription and protein expression of each virus (Zhang et al., 2007; Ormsby et al., 2012). *In vitro* studies showed that after infection with HIV-1, the transcription level of intracellular HERV-K in Jurkat T cells and primary lymphocytes was upregulated almost 20-fold compared to the control group (Gonzalez-Hernandez et al., 2012). When HIV-1 Tat protein was the only variable, HERV-K was also significantly upregulated in Jurkat T cells and primary lymphocytes (Rautonen et al., 1994; Gonzalez-Hernandez et al., 2012, 2014; Uleri et al., 2014; Young et al., 2018). This indicates that not only can the HIV-1 virus upregulate the transcription level of HERV-K in lymphocytes, but its Tat protein is also likely to play an important role in activating the transcriptional expression of HERV-K (Jang et al., 1992; Demarchi et al., 1996; Gonzalez-Hernandez et al., 2012; Uleri et al., 2014). It was reported that the increase in HERV-K (HML-2) in LC5 (a derivative of HeLa cells) and KE37.1 (T-lymphoma cells) cells during *de novo* HIV-1 infection was more obvious than that in persistent HIV-1 infection *in vitro* (Vincendeau et al., 2015). However, the degree of increase of global HERV-K (HML-2) elements was quite different, even though some elements had a downward trend. Subsequently, it was indicated that *in vitro* experiments simulating physiological infectious doses, the expression level of global HERV-K (HML-2) in cell lines did not change significantly, but some individual HML-2 elements were significantly upregulated (Vincendeau et al., 2015; Young et al., 2018; Gray et al., 2019). This suggests that HIV-1 infection may have a selective effect on the expression of HERV-K (HML-2) in cells, and the expression of each element may vary with the infectious dose and time of infection (Contreras-Galindo et al., 2017; O'Carroll et al., 2020). In addition, as a retrovirus, HIV evolves rapidly and has comprehensive diversity. Currently, the major source of the global pandemic is divided into four groups: M (Major), O (Outlier), N (Non-Mand Non-O), and P. The M group includes nine major subtypes (i.e., A-D, F-H, J, and K), over 110 circulating recombinant forms (CRFs), and numerous unique recombinant forms (URFs) (Li et al., 2020). Their comprehensive diversity makes them a serious challenge for pathogenesis research, epidemiological surveillance, diagnosis, and clinical management of infected persons. Many HIV-1 pathogenesis studies based on subtype B virus have demonstrated an underestimation of some non-B subtypes (Kline et al., 2002; Obaro et al., 2005; Buonaguro et al., 2007; Church et al., 2011). In contrast to the Americas, where HIV-1 B strains have widely spread, CRF07_BC (41.3%), CRF01_AE (32.7%), CRF08_BC (11.3%), and B (4.0%) are the predominant strains in China (Ping, 2019). Under these circumstances, a study on the correlation between HERV-K activity and HIV diversity is necessary.

Therefore, we first classified 96 HIV-infected patients according to their infection subtypes or viral load and statistically analyzed whether there were differences in HERV-K transcription levels among different groups. Next, we selected a variety of cell lines infected by different HIV-1 subtype strains and investigated the expression of HERV-K (HML-2). After that step, the cell lines with significant upregulation of global HERV-K (HML-2) were selected. The dynamic change curves of HERV-K (HML-2) elements were analyzed. We also constructed two cell lines with stable and regulated expression of HIV-1 Tat proteins originating from different subtypes. This study provides good knowledge of the effect of HIV-1 infection on the transcription of HERV-K (HML-2).

## Materials and Methods

### Patients and Samples

Blood samples were collected from 96 HIV-1-infected individuals in Anhui, Hebei, and Henan provinces in China. Among them, 39 were subtype B, 24 were CRF01_AE, 23 were CRF07_BC, and the rest were other CRFs or URFs. All HIV antibody positivity was confirmed by Western blot assay, and blood samples were collected at naïve to antiviral treatment. The healthy control group was composed of 64 blood donors with a median age of 25 years old and a male to female ratio of 1:1. None of them had a history of tumors, autoimmune diseases, or neurological diseases. All study participants provided informed consent for the collection of blood samples and subsequent analyses. All samples were transported to our laboratory through a cold chain and stored at −80°C.

### Cell Culture

The cell lines MT2, H9, 293T (without CD4 receptors), and TZM-bl (Helas) were cultured in RPMI 1640 or DMEM (Gibco, USA) with 10% fetal calf serum (Gibco, USA), 100 U/ml penicillin and 100 μg/ml streptomycin at 37°C with 5% CO_2_ and saturated humidified. All cell lines were infected (MOI = 0.1) with HIV-1 IIIB, HIV-1 NL4-3, or HIV-1 CRF01_AE, which are the widely circulating wild-type strains in China (GX002, accession: GU564222), and simultaneously parallel blank control groups (uninfected groups) were set up. The cells and cell supernatant were extracted at different time points (12, 24, and 48 h) for subsequent experiments. All cell lines and HIV-1 strains were provided by the Department of AIDS Research, Beijing Institute of Microbiology and Epidemiology.

### p24 Antigen Analysis

A total of 200 μl of cell supernatant in each group was collected separately and stored at −20°C for subsequent detection of p24 antigen. Quantification of p24 antigen in the supernatant was performed using the HIV-1 p24 antigen ELISA kit (Biomedical Engineering Center, Hebei Medical University, China) according to the manufacturer's protocol.

### Quantification of HERV-K (*env*) and HIV-1 (*gag-pol*) Transcription in Cells by RNAscope ISH Technology

RNAscope^®^ (RNAscope^®^ Fluorescent Multiplexed reagent kit, Advanced Cell Diagnostics, USA) was used according to the manufacturer's protocol and adjusted for dual detection of mRNA. The probe sets, HERV-K*env*-C1 or HIV-1 *gag*-*pol*-C2 (Advanced Cell Diagnostics, USA), consisted of 20 dual probes targeting different segments within the HERV-K-*env* or HIV-1-*gag-pol* region. After activation, in a 50 ml polypropylene centrifuge tube, the cells were collected by centrifugation at 250 RCF at room temperature for 10 min, the supernatant was aspirated, and the cells were washed twice with 10 ml of 1X PBS. The PBS was removed without touching the cell mass, leaving as little fluid as possible. Cells were immobilized in a water bath at 37°C with 10% NBF for 1 h and washed two times for 2 min with PBS. The immobilized cells were stored in 1 ml of 70% alcohol. One milliliter (10^6^) of cell fluid was placed on a glass slide and dried at room temperature for 20 min. Pretreatment for storage was performed by dehydration using 50, 70, and 100% ethanol for 5 min at room temperature. The slides were removed from anhydrous ethanol and dried at 37°C for 30 min, and a hydrophobic barrier was created by using an Immedge^™^ Hydrophobic Barrier Pen (Vector Laboratory, USA). The cells were then incubated with hydrogen peroxide and protease for 10 min and 30 min at 40°C, respectively. Probe hybridization was achieved by incubation of mixed target probes for 2 h at 40°C by using a HyBez^™^ oven. The signal was amplified by subsequent incubation of AMP-1, AMP-2, and AMP-3 one drop each for 30, 30, and 15 min, respectively at 40°C by using a HyBez^™^ oven. Each incubation step was followed by two 2 min washes using RNAscope washing buffer (Advanced Cell Diagnostics, USA). Each target nucleic acid was fluorescence-stained with TSA^®^Plus FITC and TSA^®^Plus Cy3 for 30 min at 40°C and washed twice with washing buffer. The nuclei were stained with DAPI (Advanced Cell Diagnostics, USA) for 30 s. Prolong Gold Antifade Mountant (Prolong^™^, Invitrogen) was used to prevent fluorescence quenching. Then, the coverslips were covered, and a picture was taken as soon as possible. The excitation/emission and pass wavelengths used to detect DAPI, FITC, and Cy3 were set to 340-370/410-470, 460-480/490-530, and 510-550/570-590 nm, respectively. Super-resolution images were captured using a fluorescence wide-field microscope (EVOS^™^ M5000, 10X LWD, 0.30).

### Construction of Lentivirus Tat (pNL4-3 and pGX002) With the pLVX-puro Plasmid

The Tat protein gene sequences (exon 1 and exon 2) were derived from HIV-1 strains pNL4-3 and pGX002 by specific primers. After reverse transcription, the Tat DNAs were amplified by PCR from cDNA. The amplified DNAs were ligated into the lentiviral plasmid pLVX-Tight-Puro (TaKaRa Cat No. 632162). Following transduction of DH5α-competent E. coli, the positive clones were selected by PCR identification and DNA sequencing. Clones containing the target plasmids were selected and named pLVX-Tat-NL4-3 and pLVX-Tat-GX002. At the same time, a blank plasmid (pLVX-puro) was constructed as a negative control. The plasmids were highly purified and extracted with an endotoxin-free solution. The Tat expression plasmids and pLVX-Tet-On (TaKaRa, Cat No. 632162) were then cotransfected into 293T cells with pLp1, plp2, and VSV-G using Lipofectamine^®^ 2000 (ViraPower^™^, Invitrogen) according to the manufacturers' instructions. After culturing for 48 h, the supernatant containing lentivirus particles was collected. The p24 antigen was used to determine the titer of the lentivirus (data not shown).

### Luciferase Assays

We used the TZM-bl luciferase reporter cell line to verify whether the lentivirus expressed Tat protein. TZM-bl cells were added to 96-well plates (Nunclon^™^, Thermo) at 10^4^ (60 μl) per well, and then the three lentiviruses (pLVX-NL4-3-Tat, pLVX-GX002-Tat, and pLVX-puro) were added to different wells at 40 μl per well. Each lentivirus was repeated in at least 3 wells, and a blank control (with 40 μl medium per well) was generated at the same time. After incubation at 37°C for 48 h, 20 μl per well of the luciferase assay system (Bright-Glo^™^, Promega) was added to the wells, and fluorescence detection was performed using a multimode plate reader (EnSpire, PerkinElmer) after 5 min of darkness (data not shown).

### Creating Stable Tet-On Advanced Inducible MT2 Cell Lines

The MT2 cell line was transfected with pLVX-Tat-NL4-3, pLVX-Tat-GX002, or pLVX-puro lentivirus. Lentiviruses were used at a p24 value of 30 ng/ml to infect MT2 cells. After 2 days, the infected cells were selected using 2.5 μg/ml puromycin for 1 week. Monoclonal infected cell lines were selected using limited dilution and were continuously cultured in 1640 medium at 2.5 μg/ml in 96-well plates for ~20 days. The selected monoclonal cell lines were gradually expanded to a cell population of 4 × 10^5^/ml. Then, the cell lines were replaced in a new 6-well plate with fresh culture medium, and lentivirus (pLVX-Tet-On) was added to the duplicate wells at a p24 value of 30 ng/ml. After 48 h, doxycycline (Dox) was added to the wells at a concentration of 10^3^ ng/ml. Dox (-) control groups were also created. Stable transfection was further confirmed by real-time quantitative PCR and Western blot analysis.

### RNA Preparation

Total RNA was extracted using an RNeasy Plus Mini Kit (Qiagen NO. 74134) according to the manufacturer's protocol. To remove genomic DNA, all RNA samples were treated with 1 μl of gDNA eraser per 1 μg of RNA (Takara, RR047A). Then, all RNA samples were detected for DNA contamination by real-time quantitative PCR, and the results showed that the CT value of β-actin in all samples was >40. To further assess whether these gDNA eraser-treated samples had any residual DNase activity or might inhibit PCR, we spiked treated samples or pure water controls with 10^3^ and 10^2^ DNA standards and compared their CT values. We also performed a double gDNA eraser treatment on the low-copy RNA standards, and we found no evidence of loss of RNA copies, as the CT values in successive treatments did not change (Hurst et al., 2016; Karamitros et al., 2016). Then, the mRNA was reverse transcribed into cDNA by using a mixed primer pair that contained Oligo dT primer according to the manufacturer's protocol (Takara, RR047A).

### Quantification of HERV-K, HIV-1 *pol*, and Tat Transcription in MT2 by qPCR

qPCR was performed with a Roche LightCycler 480 II System using TB Green^®^ Premix Ex Taq^™^ (TaKaRa, Cat No. RR420A). Cycling conditions were a 10-min denaturation step at 95°C, followed by 40 cycles of 10 s at 95°C, 5 s at 60°C, and 10 s at 72°C, and then a melting curve analysis to confirm the specificity of the PCR. The CT value was corrected by the corresponding β-actin control CT values. The absolute quantification of HIV-1 *pol* proviral copies was based on existing standard curves. Primer information is shown in Table 1.

**Table 1:** Primer sequences for real-time quantitative PCR.

**Primers**	**Primer sequence (5'-3')**	**Direction**
β-actin F	CCACGAAACTACGTTCAACTCC	Forward
β-actin R	GTGATCTCCTTCTGCATCCTGT	Reverse
HERV-K *gag* F	GGCCATCAGAGTCTAAACCACG	Forward
HERV-K *gag* R	CTGACTTTCTGGGGGTGGCCG	Reverse
HERV-K *pol* F	TCACATGGAAACAGGCAAAA	Forward
HERV-K *pol* R	AGGTACATGCGTGACATCCA	Reverse
HERV-K *env* F	CTGAGGCAATTGCAGGAGTT	Forward
HERV-K *env* R	GCTGTCTCTTCGGAGCTGTT	Reverse
HIV-1 *pol* F	CCAAAGTAGCATGACAAAAATC	Forward
HIV-1 *pol* R	GTTCATAACCCATCCAAAGGAATGGAGG	Reverse
Tat F	GAAGCATCCAGGAAGTCAGC	Forward
Tat R	CTTCCTGCCATAGGAGATGC	Reverse

### Western Blot Analysis

Cells were lysed in RIPA buffer containing 1% PMSF. Proteins were separated by 15% polyacrylamide gels and transferred to polyvinylidene difluoride (PVDF) membranes. Tat was detected using HIV-1 Tat monoclonal antibody (Thermo Fisher. Cat No. MA1-71509). The horseradish peroxidase-conjugated secondary antibody was α-mouse from Abcam (Cat No. ab205719).

### Statistical Analysis

The data are expressed as the mean ± s.d. taken from at least three independent experiments and compared with unpaired Student's *t*-test where appropriate using SPSS statistics 25.0 software. Correlations were assessed by using the Pearson correlation analysis. All statistical analyses were performed based on a two-sided *α* of 0.05.

### Ethics and Biosecurity Statements

Written informed consent was obtained from all HIV-positive participants and healthy blood donors, and the data were analyzed anonymously. This research project has been approved by the Ethical Board of the Beijing Institute of Microbiology and Epidemiology. The study was approved by the Biosecurity Board of the Beijing Institute of Microbiology and Epidemiology. All biological experiments involving infectious HIV-1 were conducted in BSL-3 laboratories.

## Results

### Differences in the Transcription Levels of HERV-K in Various HIV-1 Subtype-Infected Patients

**Figure 1:**
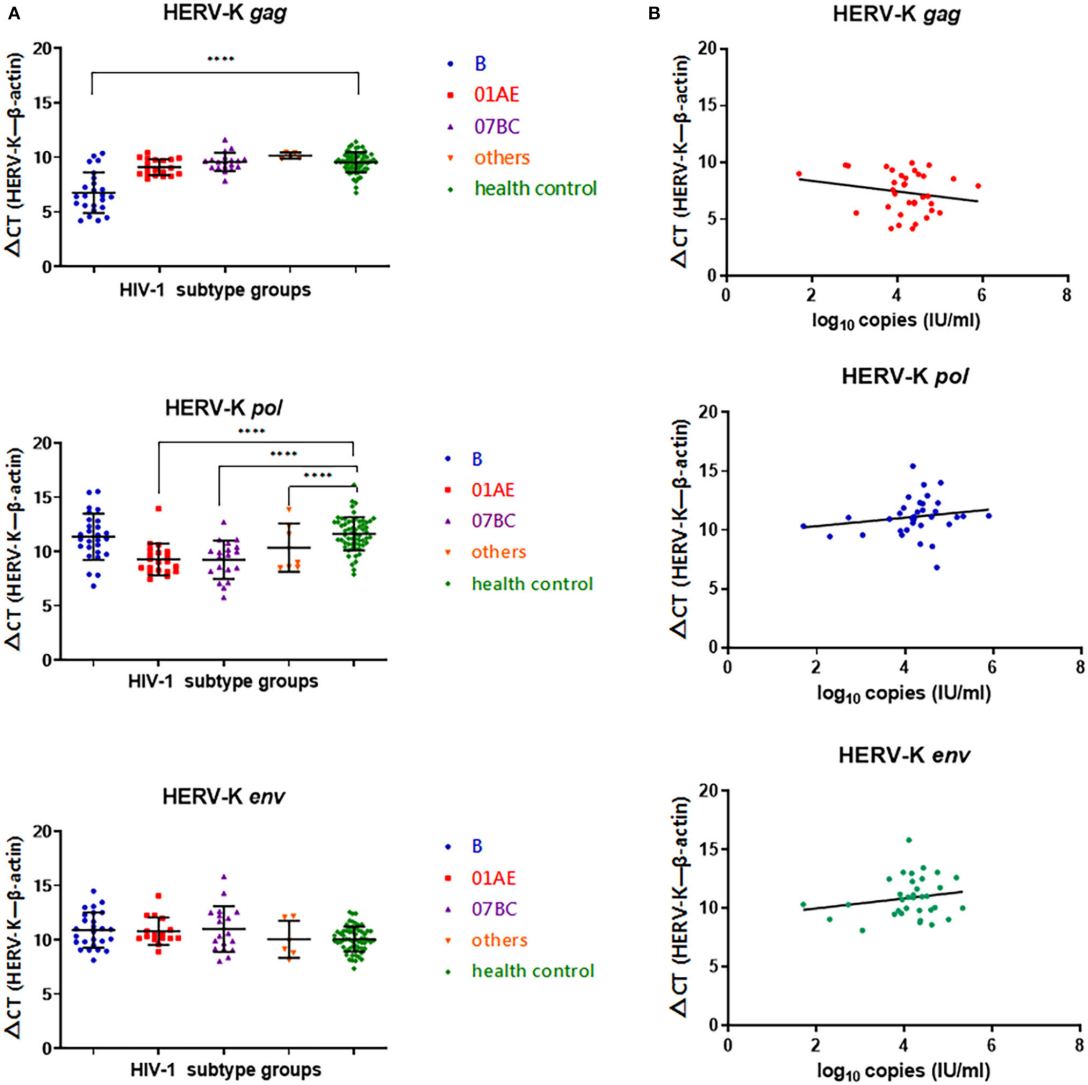
**(A)** Blue represents HIV-1B subtype-infected persons, red represents HIV-1 CRF01_AE subtype-infected persons, purple represents HIV-1 CRF07_BC-infected persons, yellow represents other CRFs and URFS-infected persons, and green represents the healthy population control group. The Y-axis is ΔCT = CT_HERV−K_−CT_β−*actin*_. ^*^P < 0.05, ^**^P < 0.01, ^***^P < 0.001, ^****^P < 0.0001. **(B)** Red represents transcriptional levels in the HERV-K *gag* region, blue represents transcriptional levels in the HERV-K *pol* region, and green represents transcriptional levels in the HERV-K *env* region. The Y-axis is ΔCT = CT_HERV−K_−CT_β−*actin*_. The X-axis represents log^10^ of the viral load in each infected person. Connecting curves are indicated.

In the HIV-1 subtype B-infected group, the ΔCT value (CT_HERV−K*gag*_−CT_β−*actin*_) of HERV-K (HML-2) *gag* was significantly lower than that of the healthy control group (*P* < 0.0001), indicating that the *gag* region of HERV-K (HML-2) in subtype B-infected patients was significantly upregulated compared with that in the healthy control group. There were no significant differences between the CRF01_AE, CRF07_BC, or other subtypes and the healthy control group (*P* > 0.05). There was no differences in the ΔCT(CT_HERV−Kpol_−CT_β−*actin*_) value of HERV-K (HML-2) *pol* region transcription between the HIV-1 B subtype-infected group and the healthy control group (*P* > 0.05). The transcriptional level of the HERV-K (HML-2) *pol* region in the CRF01_AE and CRF07_BC groups was significantly upregulated compared with that in the healthy group (*P* < 0.0001). There were no significant differences in the transcriptional level of the HERV-K (HML-2) *env* region among subtypes B, CRF01_AE, CRF07_BC, and others (*P* > 0.05) (Figure 1A).

No correlation was found between HIV-1 viral load and the transcriptional levels of HERV-K *gag* (*r* = −0.1930, *P* = 0.2668), *pol* (*r* = 0.1779, *P* = 0.3140), and *env* (*r* = 0.1878, *P* = 0.2875) in infected patient PBMCs (Figure 1B).

### Transcriptional Levels of HERV-K (HML-2) in HIV-1-Infected Cell Lines Were Different

**Figure 2:**
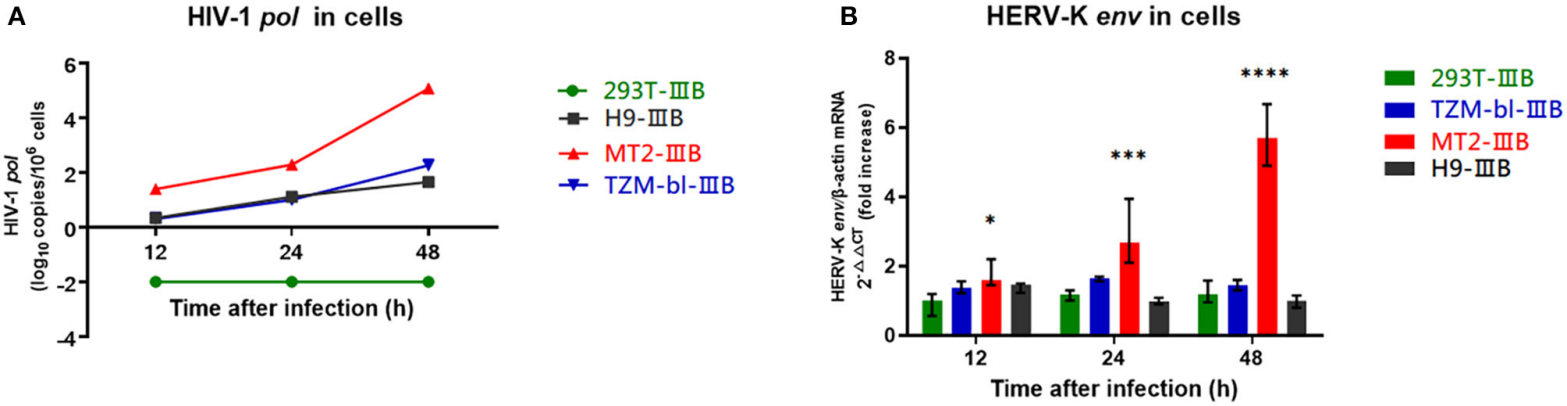
**(A)** Red represents MT2 cells, blue represents TZM-bl cells, black represents H9 cells, and green represents 293T cells. The X-axis is the time of infection. The Y-axis represents log^10^ of the HIV-1 *pol* load, and the units are indicated in the title of the Y-axis. Connecting curves are indicated. **(B)** Red represents MT2 cells, blue represents TZM-bl cells, black represents H9 cells, and green represents 293T cells. The X-axis is the time of infection. The Y-axis shows the fold increase in the transcription level in the HERV-K *env* region calculated by the 2^−ΔΔCT^ method. Standard errors for triplicate experiments are indicated. ^*^P < 0.05, ^**^P < 0.01, ^***^P < 0.001, ^****^P < 0.0001. All comparisons were relative to uninfected control cells.

MT2, TZM-bl, H9, and 293T cells were infected with HIV-1 IIIB for 48 h (MOI = 0.1). The HIV-1 viral load within MT2, H9, and TZM-bl cells increased gradually, indicating that the three cells were successfully infected. 293T cells failed to infect due to a lack of CD4 receptors (Figure 2A). The transcriptional levels of HERV-K in MT2 cells were significantly upregulated ~5 times relative to the uninfected group. In TZM-bl cells, HERV-K was only upregulated approximately twice as much as that in the uninfected group. There was no significant difference in infected H9 cells relative to the uninfected group. We also included a control group of 293T cell lines and found no statistically significant changes in HERV-K expression levels (*P* > 0.05) (Figure 2B). Therefore, we believe that HIV-1 can activate the transcription of HERV-K (HML-2) in MT2 cell lines within 48 h. In this instance, we assume that the transcription of HERV-K (HML-2) is upregulated in MT2 cells within a short time following infection. HIV-1-infected MT2 cells can be used in further studies investigating the transcription of HERV-K at different stages of HIV-1 infection.

### Transcriptional Levels of HERV-K Were Increased in HIV-1-Infected MT2 Cells Assessed by the RNAscope ISH Technique

**Figure 3:**
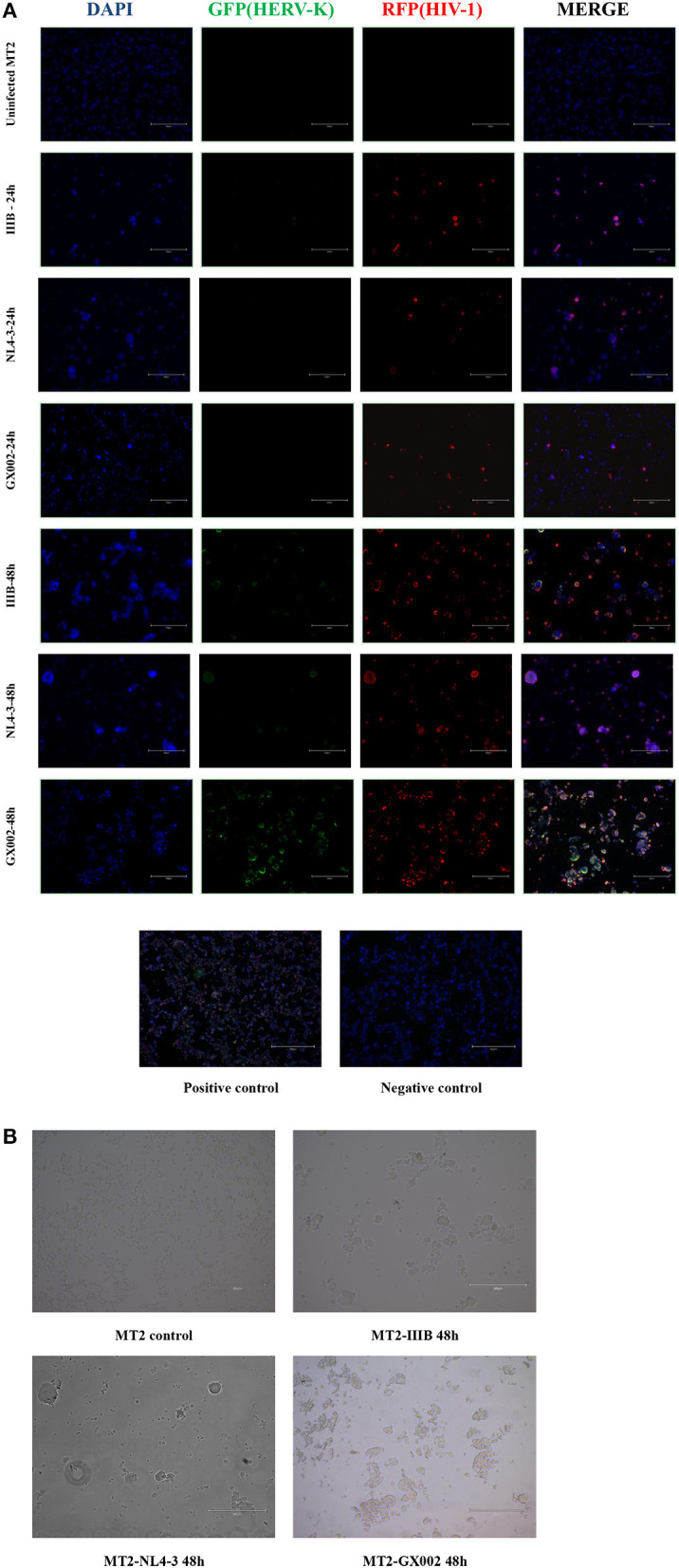
**(A)** DAPI was used to stain and label the nuclei, green fluorescent proteins (GFP) with a special probe were used to stain and label HEVR-K *env* region mRNA, and red fluorescent proteins (RFPs) with a special probe were used to stain and label HIV-1 *gag-pol* region mRNA. The positive and negative controls used standard control probes provided in the kit (ACD). **(B)** MT2 cells in both the IIIB group and GX002 group exhibited notable cytopathic effects (CPEs) after 48 h of infection. The microscopic view of uninfected MT2 cells is shown.

Sections of HIV-1-infected MT2 cells were prepared at 24 and 48 h after infection. Sections of the positive and negative controls were prepared at the same time. The proportion of HIV-1 transcription in both the IIIB-, NL4-3- and GX002-infected groups was relatively low 24 h after infection, and the transcription of HERV-K was also very low. However, at 48 h after infection, both IIIB- and GX002-infected groups showed significant increases in the transcriptional levels of HIV-1 and HERV-K, and the GX002 group had higher levels of HERV-K transcription than the IIIB-infected group (Figure 3A). HIV-1 transcription occurred in ~90% of the cells after 48 h of infection, as indicated by MERGE graphs. MT2 cells had notable cytopathic effects (CPEs) (Figure 3B). Therefore, we could not use flow cytometry to screen infected cells for subsequent real-time quantitative PCR. This helped us avoid the adverse effects of flow cytometry on cells and retain more cells for subsequent experiments.

### HIV-1 IIIB, NL4-3, and CRF01_AE Strains Lead to Differences in HERV-K Transcription Levels in MT2 Cells

**Figure 4:**
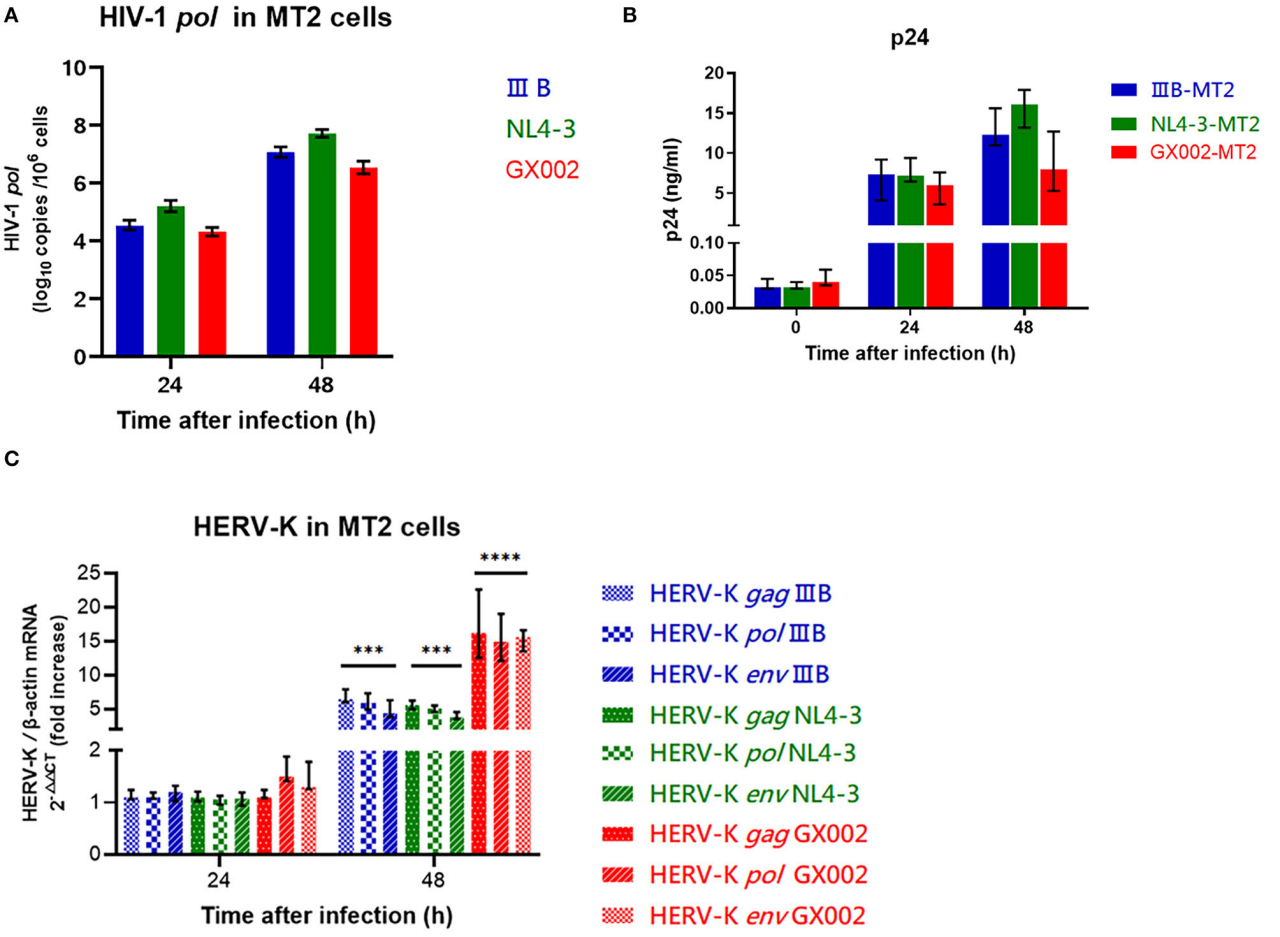
**(A)** Red represents the GX002 group, blue represents the IIB group, green represents the NL4-3 group. The X-axis is the time of infection. The Y-axis represents log^10^ of the HIV-1 *pol* load, and the units are indicated in the title of the Y-axis. Standard errors for triplicate experiments are indicated. **(B)** Red represents the GX002 group, blue represents the IIB group, green represents the NL4-3 group. The X-axis is the time of infection. The Y-axis represents the value of p24 antigen, and the units are indicated in the title of the Y-axis. Standard errors for triplicate experiments are indicated. **(C)** The bars represent the groups indicated in the figure. The X-axis is the time of infection. The Y-axis shows the fold increase in transcription level in the HERV-K *gag, pol, env* region calculated by the 2^−ΔΔCT^ method. Standard errors for triplicate experiments are indicated. ^*^P < 0.05, ^**^P < 0.01, ^***^P < 0.001, ^****^P < 0.0001. All comparisons were relative to uninfected control cells.

The wild-type HIV-1 CRF01_AE strain (GX002), which is widely prevalent in China, and the HIV-1 IIIB strain HIV-1 NL4-3 strain were used to infect MT2 cells (MOI = 0.1). After infection, GX002 replicated less efficiently in infected MT2 cells than IIIB and NL4-3 when comparing the HIV-1 load within cells and the concentration of p24 antigen in the supernatant (Figures 4A,B). However, the fold increase in the level of HERV-K transcription (2^−ΔΔCT^) in MT2 cells was higher in the GX002-infected group than in the IIIB-infected and NL4-3-infected groups. Compared to the uninfected group was 15-fold higher in the GX002 group, 5-fold higher in the IIIB and NL4-3 groups, respectively (Figure 4C).

### Tet-On System Successfully Regulated the Protein Expression of the Tat Gene

**Figure 5:**
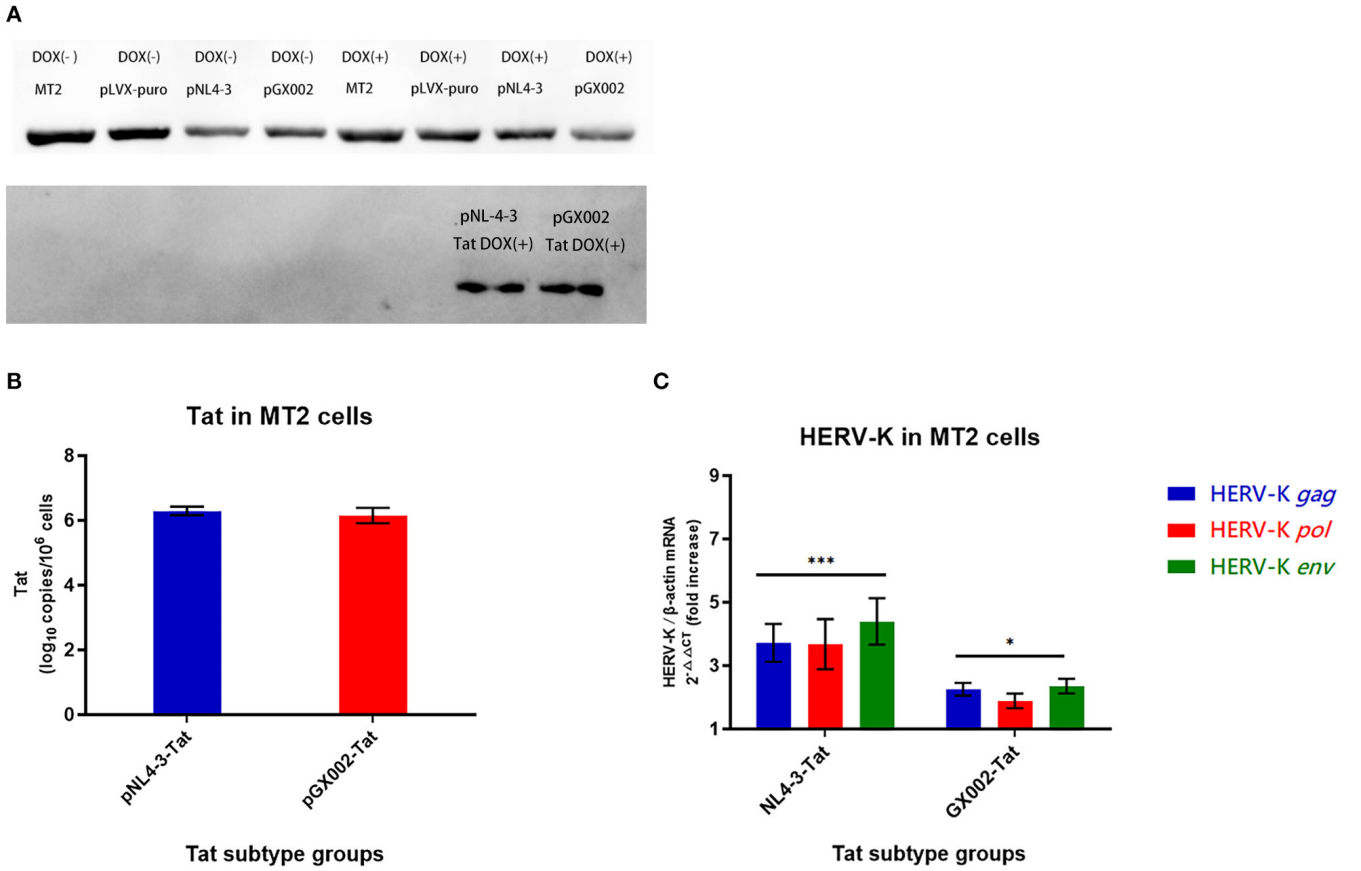
**(A)** pNL4-3 and pGX002 in the DOX(+) groups expressed large amounts of Tat protein; MT2 cells in the uninfected group and the blank plasmid-transfected group DOX(+) did not express Tat protein; all MT2 cells in the DOX(-) groups did not express Tat protein. **(B)** Red represents the pGX002 group, blue represents the pNL4-3 group. The Y-axis represents log^10^ of the Tat gene load, and the units are indicated in the title of the Y-axis. Standard errors for triplicate experiments are indicated. **(C)** Blue represents HERV-K *gag*, red represents HERV-K *pol*, green represents HERV-K *env*. The X-axis is Tat subtype groups. The Y-axis shows the fold increase in transcription level in the HERV-K *gag, pol, env* region calculated by the 2^−ΔΔCT^ method. Standard errors for triplicate experiments are indicated. ^*^P < 0.05, ^**^P < 0.01, ^***^P < 0.001.

In this study, an induced Tat expression system was constructed by using an optimized Tet-On vector. An MT2 cell line in which the Tat proteins of type B and CRF01_AE were stably expressed was constructed for the first time. Tat-positive cells could be screened highly efficiently by coexpressing the puromycin resistance gene with Tat. Finally, two stable Tat expression cell lines (pNL4-3-Tat and pGX002-Tat) were successfully obtained. The control groups for Dox (-) were also set up. Dox induction was performed on two Tat expression cell lines and the control group mentioned above. Western blot analysis showed that the Tat protein (pNL4-3-Tat, pGX002-Tat) was significantly expressed in the two groups of cells treated with Dox (10^3^ ng/ml) for 72 h (Figure 5A).

### Transcription of HERV-K (HML-2) and Expression of Tat Among Cell Lines Analyzed by qPCR

High levels of Tat protein gene expression were detected in two pLVX-Tat cell lines (pLVX-NL4-3-Tat and pLVX-GX002-Tat), indicating that monoclonal cell screening was successful using culture medium containing puromycin (Figure 5B). The influence of Tat expression on HERV-K (HML-2) transcription in MT2 cells was detected by real-time quantitative PCR. Both pNL4-3-Tat and pGX002-Tat led to an increase in HERV-K (HML-2) transcription. In addition, pNL4-3-Tat had a stronger effect on HERV-K (HML-2) than pGX002-Tat. Compared with the blank control, the transcriptional levels of HERV-K (HML-2) were increased ~4-fold in the pNL4-3 group (*P* < 0.001) and 2-fold in the pGX002 group (*P* < 0.05) (Figure 5C).

## Discussion

HERV-K (HML-2) contains the most active retrovirus in the human genome and maintains open reading frames encoding functional viral proteins that are expressed but form noninfectious particles (Büscher et al., 2005; Subramanian et al., 2011; Schmitt et al., 2013; White et al., 2018). Many studies have reported that HIV-1 infection is closely related to upregulation of HERV-K (HML-2) expression (Contreras-Galindo et al., 2006; Ormsby et al., 2012; Vincendeau et al., 2015; Monde et al., 2017; Young et al., 2018; Su et al., 2019; Srinivasachar Badarinarayan et al., 2020). Although the mechanism underlying this effect is still unclear, many studies have shown that the Tat and Vif proteins of HIV-1 are involved in the upregulated expression of HERV-K (HML-2) (Gonzalez-Hernandez et al., 2012, 2014; Jones et al., 2012; Young et al., 2018; Srinivasachar Badarinarayan et al., 2020). In this study, the ΔCT = CT_HERV−Kgag_−CT_β−*actin*_ value of PBMCs collected from HIV-1 subtype B-infected patients was lower than that of PBMCs collected from healthy controls, indicating that the transcription level of HERV-K (HML-2) *gag* was significantly upregulated in HIV-1 subtype B-infected individuals. However, the transcription level of HERV-K (HML-2) *gag* in PBMCs of the CRF01_AE- and CRF07_BC-infected groups was not significantly upregulated. The transcription level of the HERV-K (HML-2) *pol* region in the CRF01_AE- and CRF07_BC-infected groups was significantly upregulated compared with the healthy control group, but there was no significant difference in the subtype B-infected group; No significant difference was found in the transcriptional levels of the *env* region of HERV-K (HML-2) in the diverse subtype-infected groups compared with healthy controls. It was suggested that there are subtype differences in the activation of HERV-K (HML-2) transcription between subtype B and non-subtype B-infected groups (CRF01_AE, CRF07BC), and there are differences in the activation regions of HERV-K (HML-2). To explain these differences, we compared the genetic sequences of the HIV-1 subtypes. The similarity of the NFLG sequence between CRF01_AE and subtype B was 83.5%, the similarities of Tat-exon1 and Tat-exon2 were 79.5 and 85.9%, respectively, the similarity of Vif was 83.2%, and the similarities of Rev-exon1 and Rev-exon2 were 75 and 74.7%, respectively. The similarity of the NFLG sequence between CRF07_BC and subtype B was 85.6%, the similarities of Tat-exon1 and Tat-exon2 were 86.6 and 82.9%, respectively, the similarity of Vif was 85.2%, and the similarities of Rev-exon1 and Rev-exon2 were 80.3 and 73.5%, respectively (https://www.ebi.ac.uk/Tools/psa/emboss_water/). Diverse HIV-1 subtypes exhibit notable differences in gene homology, which may be an important reason for the differences in the transcription levels of HERV-K (HML-2) activated by HIV-1 infection. Second, all blood samples we tested were collected from HIV-1-infected patients without antiviral treatment, and the effect of antiviral drugs on the transcription of HIV-1 and HERV-K (HML-2) was excluded. However, most of the subtype B patients in the study were infected through paid blood donation in the 1990's, and their HIV-1 viral load might have been higher in the early acute stages of infection compared with those patients who were infected through sexual transmission. As a result, the transcriptional level of HERV-K (HML-2) was affected by a relatively high concentration of HIV-1, resulting in significant upregulation of transcription in the *gag* region, which is closer to the HERV-K (HML-2) LTR. Most CRF01_AE-, CRF07_BC- and other subtype-infected persons were infected *via* sexual transmission, and the viral load of HIV-1 in the early acute stage of infection was relatively low. During the process of viral replication and proliferation, the activation of the *gag* regions of HERV-K (HML-2) may be inhibited by the immune system, while the *pol* regions of HERV-K (HML-2) are activated because they are farther away from the LTR region. Therefore, we analyzed the correlation between viral load and HERV-K (HML-2) transcription levels in infected patients. No correlation was found between HERV-K (HML-2) transcriptional levels and HIV-1 viral load. However, this is only a prediction based on the current patients and limited viral load tests and cannot fully explain how the differences in HIV subtypes lead to differences in the activation of HERV-K (HML-2) in different regions. It is necessary to further expand the sample size to study the effects of diverse HIV-1 subtype infections on the activation of HERV-K (HML-2) transcription.

CD4 and CCR5/CXCR4 molecules on the surface of target cells are used by HIV-1 as receptors and coreceptors, respectively. However, even in the presence of receptors and coreceptors, different subtypes of HIV-1 strains differ greatly in their susceptibility to cells (Fantuzzi et al., 2003; Abraha et al., 2009; Singh et al., 2014). In the *in vitro* experiment, we did not simulate the natural infectious dose of HIV-1 but used a relatively higher infectious dose (MOI = 0.1). We hoped that more cells could be infected as soon as possible with the premise of ensuring the normal survival of cells within 48 h. MT2 cells derived from T lymphocytes were more susceptible to two subtypes of HIV-1 strains within 48 h, and the level of HERV-K (HML-2) transcription in MT2 was upregulated after infection. The number of T lymphocytes is relatively small in the peripheral blood, and the viral load in most infected people is relatively low. The results of the *in vitro* experimental study were not completely consistent with the results of the *in vivo* study. Before using RT-qPCR to detect the transcription level of HERV-K, RNAscope was used to fluorescently label the specific target mRNA of infected cells. The infection of MT2 cells and the change in HERV-K transcription level in cells can be observed more intuitively by fluorescence microscopy. RNAscope uses software to calculate the proportion of HIV-infected cells and determine whether HIV-infected cells need to be screened by flow cytometry to eliminate interference from uninfected cells. The RNAscope technique has the advantage of having a lower limit of detection (LOD) for testing latent HIV infection compared to flow cytometry (Zhang et al., 2018). Combined with the results of real-time quantitative tests, we found that CRF01_AE strains, which are widely prevalent in China, have a stronger ability than laboratory-adapted IIIB and NL4-3 strains to activate HERV-K transcription. GX002 had a 15-fold increase compared to the blank control, while IIIB and NL4-3 groups had an ~5-fold increase. However, strain IIIB and NL4-3 possessed greater fitness to infect and replicate in MT2 cells, which was reflected in a load of HIV-1 *pol* within the cell and the concentration of p24 antigen in the supernatant. Therefore, it is not the case that a higher level of HIV strain replication results in stronger activation of HERV-K (HML-2). These results suggested that there might be biological elements in the CRF01_AE (GX002) genome that is more likely to activate HERV-K (HML-2). We first considered whether the Tat proteins of the two HIV-1 subtype strains differed in activating HERV-K (HML-2) transcription. A large number of reports have shown that the Tat protein plays an important role in the activation of HERV-K (HML-2) transcription (Gonzalez-Hernandez et al., 2012, 2014; Young et al., 2018). The similarity of NFLG sequences between CRF01_AE and subtype B was 84.5%, the similarities of Tat-exon 1 and Tat-exon 2 were 80.9 and 76.8%, respectively, and the Tat sequences of these two strains varied widely. Therefore, we designed the next study to explore the differences in the activation of HERV-K (HML-2) transcription by the Tat protein of HIV-1 subtype B and CRF01_AE.

HIV-1 Tat is an effective transactivator of the HIV-1 promoter, which is essential for HIV-1 replication and antigen expression (Jang et al., 1992). In addition to activating HIV-1 transcription in cells, Tat also acts on other viral and cellular promoters (Bohan et al., 1992; Gonzalez-Hernandez et al., 2012). Both HIV-1 and HERVS belong to retroviruses, which have similar genomic structures and encode similar proteins. The Tat protein binds to the LTR of HERVS and promotes the transcription of HERVS and its downstream genes. The effect of Tat on the expression of HERV-K (HML-2) occurred at the transcriptional promotion level (Gonzalez-Hernandez et al., 2012). In this study, we successfully constructed an MT2 cell model that can regulate and stably express the Tat protein of subtype B and CRF01_AE. The results of this study provide a basis for further studies on the effect of Tat of HIV-1 subtype B and CRF01_AE on HERV-K (HML-2) transcription in MT2 cells. We found that the effect of pNL4-3 (subtype B) Tat on increasing HERV-K (HML-2) transcription in MT2 cells was more obvious than that of pGX002 (CRF01_AE) Tat, which was inconsistent with the results in Results 4 of this study. The virus strain (GX002) we used in this study was a clinical isolate of CRF01_AE, while the Tat protein of GX002 used in this study was obtained from the preserved virus strain after repeated virus rescue. During the process of virus culture and rescue, some genetic mutations may occur, which may lead to changes in the infectious capacity of the virus and the biological activity of Tat. Although the genetic homology of the two subtype B strains reached 95% and the similarities of the Tat-exon1 and Tat-exon2 genes also reached 94.9 and 91.2%, there were still a few bases and amino acid differences between the two strains. However, the Tat protein of strain IIIB has 15 and 17 fewer amino acids than NL4-3 and GX002, respectively. The missing amino acid sequences were all from Tat-Exon2 (https://www.hiv.lanl.gov/components/sequence/HIV/search/search.comp). By contrast, the Tat protein of the NL4-3 strain is more similar in size to the Tat protein of the current circulating subtype B strain. Therefore, we used the Tat protein of the NL4-3 strain. Second, although the Tat protein can stimulate the elevation of HERV-K (HML-2) transcription, it is not the only factor contributing to the activation of HERV-K (HML-2) transcription by HIV-1 infection. For example, the Vif and Vpu proteins of HIV-1 also play an important role in the activation of HERV-K (HML-2) (Gonzalez-Hernandez et al., 2012). More research is needed to determine the interactions among these proteins. Our data once again prove that the activation of HERV-K (HML-2) by HIV-1 is a very complex process, and many in-depth studies are still needed to explain the mechanism.

In conclusion, phenotypic differences were found between subtype B and the CRF01_AE strain in the activation of HERV-K. Furthermore, the activation of HERV-K (HML-2) by the Tat protein of the two strains was biologically verified. Later experiments may require transcriptome analysis of the two strains and Tat protein on CD4 T cells, which is expected to provide more clues and inspiration for a study of the interaction mechanism between HIV-1 and HERV-K (HML-2).

## Data Availability Statement

The original contributions presented in the study are included in the article/supplementary material, further inquiries can be directed to the corresponding author/s.

## Ethics Statement

The studies involving human participants were reviewed and approved by the Ethical Board of the Beijing Institute of Microbiology and Epidemiology. The patients/participants provided their written informed consent to participate in this study. Written informed consent was obtained from the individual(s) for the publication of any potentially identifiable images or data included in this article.

## Author Contributions

YG and ZP provided a lot of help in supplementing experiments and revising manuscripts. All authors contributed to the article and approved the submitted version.

## Conflict of Interest

The authors declare that the research was conducted in the absence of any commercial or financial relationships that could be construed as a potential conflict of interest.
